# Caries Lesion Assessment Using 3D Virtual Models by Examiners with Different Degrees of Clinical Experience

**DOI:** 10.3390/medicina59122157

**Published:** 2023-12-13

**Authors:** Ioana Porumb (Chifor), Daniel-Corneliu Leucuta, Marion Nigoghossian, Bogdan Culic, Patricia Ondine Lucaciu, Carina Culic, Iulia Clara Badea, Alexa-Nicole Leghezeu, Andra Gabriela Nicoara, Meda-Romana Simu

**Affiliations:** 1 Department of Preventive Dentistry, Iuliu Hatieganu University of Medicine and Pharmacy, 31 A Iancu Str., 400083 Cluj-Napoca, Romania; ioana.chifor@umfcluj.roiulia.badea@umfcluj.ro (I.C.B.); 2 SC Chifor Meddent SRL, 9 Gh Doja Str., 400068 Cluj-Napoca, Romania; 3 Department of Medical Informatics and Biostatistics, Iuliu Hatieganu University of Medicine and Pharmacy, 4 Pasteur Str., 400349 Cluj-Napoca, Romania; dleucuta@umfcluj.ro; 4 Faculty of Dental Medicine, Iuliu Hatieganu University of Medicine and Pharmacy, Avram Iancu Str., No 31, 400083 Cluj-Napoca, Romania; leghezeu.alexa.nicole@elearn.umfcluj.ro (A.-N.L.); nicoara.andra.gab@elearn.umfcluj.ro (A.G.N.); 5 Dental Propedeutics and Aesthetics Department, Iuliu Hatieganu University of Medicine and Pharmacy, 32 Clinicilor Str., 400006 Cluj-Napoca, Romania; bculic@umfcluj.ro; 6 Department of Oral Health, Iuliu Hatieganu University of Medicine and Pharmacy, 15 Victor Babes Str., 400012 Cluj-Napoca, Romania; patricia.lucaciu@umfcluj.ro; 7 Department of Odontology, Iuliu Hatieganu University of Medicine and Pharmacy, 33 Motilor Str., 400001 Cluj-Napoca, Romania; 8 Department of Pediatric Dentistry, Iuliu Hatieganu University of Medicine and Pharmacy, 400083 Cluj-Napoca, Romania; romana.simu@elearn.umfcluj.ro

**Keywords:** intraoral scanners, dental caries, diagnostic techniques, 3D virtual models

## Abstract

*Background and Objectives*: Dental caries is a preventable, reversible disease in its early stages. This study evaluated the intra-rater agreement of International Caries Assessment and Detection System (ICDAS) scores with Medit i500^®^ and Omnicam^®^ scanners versus traditional clinical examinations and the inter-rater agreement using the Omnicam^®^ among senior dentists and dental students and between these two groups. *Materials and Methods*: A total of 24 patients aged between 21 and 34 years, randomly selected from dental students and interns, underwent four examinations (three intraoral scans and one clinical examination), and the corresponding ICDAS scores were recorded by a randomly selected rater out of the 31 available examiners. The examination team consisted of dental students, dentists with less than 3 years, and dentists with more than 5 years of clinical experience. The following inter- and intra-rater agreement tests for the ordinal data were chosen: Fleiss’ kappa coefficient, Cohen’s weighted kappa, and inter-class correlations. *Results*: For all examination techniques, there was statistically significant agreement for the experienced raters (*p* < 0.05). The highest positive interclass correlation was obtained for inter-rater agreement tests of 288 observations recorded by senior dentists: ICC = 0.969 (95% CI 0.949–0.981). *Conclusions*: Intra-rater reliability was excellent for Omnicam compared to clinical exams conducted by senior dentists but moderate for Medit i500. Although inter-rater agreement using Omnicam was poor between students and between senior dentists and students, it was excellent among senior dentists.

## 1. Introduction

Dental caries are the most common oral disease. The World Health Organization (WHO) Global Oral Health Status Report from 2022 shows that nearly 2 billion people suffer from permanent tooth decay and that 514 million children suffer from primary tooth decay [1]. One significant reason explaining this high prevalence is that early-stage lesions are particularly difficult to diagnose and often remain undetected, mostly because of false negative results, and later evolve into cavities [2,3]. To prevent tooth decay, it is necessary to implement a correct diagnosis to achieve effective treatment, so early detection methods should be improved, especially since diagnosticated lesions can be treated with non-invasive therapy. This is very important, especially for early-career dental care professionals to avoid overtreatment temptations and for insurance companies to avoid unnecessary follow-ups.

There are many ways to diagnose carious lesions. The most common method used worldwide is clinical visual examination, which was described by specialists as having a high specificity and low sensitivity and reproducibility [4]. Low sensitivity comes from the inaccurate identification of carious sites, while low reproducibility arises from the subjective nature of this method, which relies heavily on the operator’s experience [5,6]. Kühnisch’s study has shown that the use of a sharp probe during the clinical examination can lead to pit and fissure damage, which can increase lesion progression, so more visual and less invasive diagnostic methods are recommended [7].

In addition to clinical examination, bitewing radiography may be used with good results for proximal caries lesion detection. However, it should not be regarded as the gold standard [8]. In their systematic review, Muñoz-Sandoval et al. concluded that additional clinical data are needed to draw a definitive conclusion on this issue [9]. Bitewing radiography is recommended as a compliment to visual inspection and produces favorable outcomes; however, it should not be regarded as the ultimate benchmark when used independently. The detection of cavities using a radiopaque dye may be also an efficient option, but it still requires the patient to be exposed to X-rays [10]. Fiber-optic transillumination (FOTI) can also be used for caries diagnoses. Carious tooth structures display fluorescence proportional to the extent of decayed tissue, and this property was the basis for the DIAGNOdent^®^ device (KaVo, Biberachl Riss, Germany) that was introduced in 1998 and has since become one of the most studied and widely used devices [11,12,13]. Most studies evaluating its specificity and sensitivity have been conducted using extracted teeth. The category of diagnosis was classified according to the range of scores: a range from 0 to 10 corresponds to the healthy tooth structure category; a range from 11 to 20 corresponds to the outer half enamel caries category; a range from 21 to 30 corresponds to the inner half enamel caries category; and a range over 30 corresponds to the dentin caries category [14]. Some authors have suggested that modifications to the manufacturer’s recommended cutoff values could improve the diagnostic efficiency of the DIAGNOdent^®^ pen [15]. In vivo and in vitro studies have chosen different cutoff values for DIAGNOdent^®^ than those recommended by the manufacturer. The optimal cutoff points for DIAGNOdent^®^ and their corresponding histological threshold values were classified as follows: sound diagnosis corresponds to a D0 threshold and values in the range of 0–13; enamel caries lesion diagnosis corresponds to a D1–D2 threshold and values in the range of 14–29; dentin caries lesion diagnosis corresponds to a D3–D4 threshold and values greater than 30 [16]. The review published in 2021 by Foros et al. suggests that clinical examination is the most effective method for early caries diagnosis in both primary and permanent teeth and can be enhanced using DIAGNOdent, especially for occlusal caries [2].

A bibliometric analysis of the state of the art in caries diagnosis has shown that commonly cited topics involve the accuracy of diagnostic techniques, concepts, and theories employed in the diagnostic procedure and the broader implications of caries diagnosis, concluding that early-stage lesion detection can still be significantly improved [17].

The identification of a carious lesion has become a lot easier thanks to the progress made in digital diagnostic technologies [18]. In this context, intraoral scanners (IOSs) that use optical impressions have evolved considerably in recent years and have shown some potential in the detection and monitoring of oral diseases. Additionally, they are becoming available for most dental clinics [19,20]. Combined with artificial intelligence, monitoring of incipient carious lesions could be greatly streamlined and used effectively in cariology. Thanks to the three-dimensional images provided by IOS, including approximate true colors, they could possibly represent a relevant tool for remote diagnosis as part of teledentistry, especially for patients whose access to preventive care services is very limited or during exceptional situations, such as the COVID-19 pandemic [20,21]. Artificial intelligence (AI) algorithms have been developed recently for this purpose and are currently being assessed [22,23]. Some have been validated, with an overall in vivo diagnostic performance comparable to that of visual examination [22]. A recent study found significant correlations between on-screen examinations, clinically recorded ICDAS scores, and histological scores [24].

To the extent of our search, no studies have addressed the influence of the examiner’s experience level on the diagnosis of caries lesions using color 3D virtual models.

This study aimed to assess the consistency of dental examination techniques, focusing on intra- and inter-rater agreements among senior dentists. This study compared intra-rater agreements using Medit i500^®^ and Omnicam^®^ scanners against clinical examinations. Additionally, we examined inter-rater agreements with the Omnicam^®^ scanner, in the following cases: among senior dentists, then among dental students, and finally between senior dentists and dental students.

## 2. Materials and Methods

### 2.1. Study Design and Setting

We conducted a prospective cohort study in the Faculty of Dentistry, ‘Iuliu Hatieganu’ University of Medicine and Pharmacy, Cluj-Napoca, Romania. This study was approved by the Ethics Committee of the “Iuliu Hatieganu” University of Medicine and Pharmacy (DEP125/20 April 2023). The participants received detailed information on our research. Signed informed consent was obtained from all participants before inclusion in this study.

### 2.2. Participants

A total of 24 volunteers (selected among the 60 interns in general dentistry and 98 third-year dental students) were selected to participate in this study. According to the available devices, at the beginning of each examination meeting, the subjects were allocated for an examination type (out of the four tested types) and an examiner among the 31 available ones grouped into three experience levels: (a) third-year dental students; (b) interns in general dentistry (1–3 years of clinical experience as cariologists); and (c) senior dentists (more than 5 years of clinical experience in cariology). The examiner was randomly selected after excluding raters who had seen the same patient (or their 3D virtual models) in the last three months.

The inclusion criteria were adult volunteers, students, and interns in general dentistry at the Department of Preventive Dentistry of Iuliu Hatieganu University of Medicine and Pharmacy, Cluj-Napoca, Romania.

Children, patients with contraindications to professional cleaning, and patients with COVID-19 were not included in this study.

Patients were prepared for this study and then examined clinically according to the ICDAS file methodology and/or paraclinically (intraoral scans).

### 2.3. Cleaning of Evaluated Teeth

Before the examination, the teeth were carefully cleaned. All teeth were cleaned using a water-powder jet cleaner, autoclavable 135° (Air flow → Handy 2+, EMS, Nyon, Switzerland) containing sodium bicarbonate powder. Powder remnants were removed by rinsing the teeth with a water spray for 5 s each.

### 2.4. Diagnostic Methods

#### 2.4.1. Visual Examination (ICDAS)

Examinations were performed in a dental office with proper illumination (25,000 lx of the dental unit lamp); an air syringe; a plane buccal mirror; and, if necessary, a WHO periodontal probe. The clinical examiner assessed all teeth in vivo on wet and air-dried surfaces for 5 s, and the lesions were classified according to the ICDAS II criteria [25,26,27], with a two-digit code as the detection criteria for primary coronal caries. The first is related to the restoration of teeth and has a code that ranges from 0 to 9. The second digit ranges from 0 to 6, and it is used for coding the caries.

A pre-calibration of all examiners was carried out as follows: training with the ICDAS clinical caries criteria and the recording of caries clinical scores were conducted by a university lecturer. The caries assessment training encompassed both a preliminary ICDAS onsite course, which specified the diagnostic criteria, and subsequent didactic hands-on training. During the hands-on training, five examinations of patients were performed, and participants evaluated teeth that presented lesions across all severity and cavitation levels.

#### 2.4.2. Intra Oral Scanners

Three IOS systems were used to scan all teeth: Medit i500^®^ (MEDIT Corp., Seoul, Republic of Korea), Virtuo Vivo^®^ by Straumann, and Omnicam^®^ (Dentsply Sirona, Charlotte, NC, USA). The scan parameters were set as suggested by the manufacturer, in blue-light mode, a filtering level of 2, and a focal length of 17 mm, in a dark environment (the dental unit light was turned off). All dental surfaces were air-dried for 5 s before scanning. Intraoral scans were performed during the same appointment as the clinical examinations.

The acquired three-dimensional color image data were visualized using the Exocad viewer software (version 1.6.2/2021), as shown in Figure S1. Like direct visual clinical examination, lesions were assessed using three-dimensional models, classified according to the ICDAS II criteria, and recorded on an ICDAS chart. There was a delay of 3 months between the clinical examinations and the ratings recorded while visualizing the 3D virtual models.

The values assigned by each examiner (rater), on each of the ICDAS-defined surfaces during examinations, were collected in a structured database built in Microsoft^®^ Excel^®^ for Microsoft 365 MSO (Version 2306 Build 16.0.16529.20164), 64-bit.

### 2.5. Missing Data

Only complete cases were included in the analysis, where the values were recorded for each ICDAS surface, and the examiner and the patient’s assigned identifier as well as the examiner’s level of expertise and examination type were clearly marked. Volunteers with missing clinical rating were excluded from all the analyses.

### 2.6. Statistical Analysis

Inter-rater and intra-rater agreements for the ordinal data were measured using Fleiss’ kappa coefficient, interclass correlation coefficient, and a 95% confidence interval. Furthermore, a formal statistical test was used to check the significance of the results.

The following agreement assessments were performed for the same level of clinical experience and different levels of clinical experience, respectively:

- Between examination techniques with the same rater (intra-rater agreement);
- Between raters for the same examination technique (inter-rater agreement).

To assess the agreement between examination techniques with the same rater, we performed the following analysis: senior dentists intra-rater agreement tests for Medit i500^®^ compared with clinical examination; senior dentist intra-rater agreement tests for Omnicam^®^ compared with clinical examination. Then, we employed inter-rater agreement tests for the same examination technique via inter-rater agreement tests for examiners with the same experience level for Omnicam (for senior dentists and dental students, respectively). We also conducted inter-rater agreement tests for examiners with different experience levels for Omnicam.

The agreement tests were performed separately for frontal (11–13, 21–23, 31–33, and 41–43) and lateral teeth (all other teeth except for the frontal teeth) due to wide differences in the visibility and difficulty levels of the examination. For the same reason, comparisons were performed between pits and fissures on smooth surfaces.

To quantify the scores for the surfaces where carious lesions were identified, the following protocol was used: caries was considered if the last digit of the ICDAS scores (clinical examinations and 3D model assessment) was ≥1 and ≤6; early stage, non-cavitary caries were considered if the last digit of the ICDAS scores was ≥1 and ≤3.

The reporting of the present study followed the EQUATOR guidelines, specifically the STROBE statement [28].

Statistical analyses were performed using the R environment for statistical computing and graphics (R Foundation for Statistical Computing, Vienna, Austria) version 4.3.1 using the irr R package [29].

## 3. Results

The study group had a similar distribution to the male/female dental student ratio: 6 males (25%) out of the 24 volunteers. Intra-rater and inter-rater agreement tests and interclass correlations showed statistically significant results (*p* < 0.001) for senior dentists.

For the 24 patients, the 31 available examiners performed both clinical and 3D virtual model ICDAS scoring, as shown in Table 1.

The available data allowed us to perform the following agreement tests.

### 3.1. Agreement Tests between Examination Techniques with the Same Rater

#### 3.1.1. Senior Dentist Intra-Rater Agreement Tests for Medit i500^®^ Compared with Clinical Examination

For the same rater (number 3, experience level c, senior dentist), the Medit i500^®^ intraoral scanner acquired 3D models that were in statistically significant agreement (*p* < 0.001) with the clinically recorded ICDAS file for a total of 287 observations (ICDAS teeth areas) in two patients (Table 2).

When intra-rater agreement was tested separately for visible (frontal) and lateral areas, as shown in Table 2, the results were statistically significantly comparable (*p* < 0.001) for the Medit i500^®^ intraoral scanner-acquired 3D model in comparison to the clinically recorded ICDAS file. For the frontal area, we obtained low positive values for Cohen’s weighted Kappa and ICC and a moderately positive value for Fleiss kappa. There was also a statistically significant (*p* < 0.001) intra-rater moderately positive agreement for the Medit i500^®^ intraoral scanner-acquired 3D model in comparison to the clinically recorded ICDAS file both for pits and fissures, as well as for smooth surfaces.

#### 3.1.2. Senior Dentist Intra-Rater Agreement Tests for Omnicam^®^ Compared with Clinical Examination

All intra-rater agreement tests for Omnicam^®^ compared with clinical examination for rater no. 3 (senior dentist) showed statistically significant values (*p* < 0.01), as shown in Table 3.

The highest inter-rater agreement result was an ICC of 0.969 for 64 observations recorded on pits and fissures by senior dentists using Omnicam^®^.

## 4. Inter-Rater Agreement Tests for the Same Examination Technique

### 4.1. Inter-Rater Agreement Tests for Examiners with the Same Experience Level for Omnicam

#### 4.1.1. Senior Dentists

For two different raters (numbers 3 and 31) with the same experience level (c = senior dentist), the Omnicam^®^ intraoral scanner-acquired 3D models were statistically significantly comparable (*p* < 0.001) for a total of 288 observations on two patients, as shown in Table 4.

#### 4.1.2. Dental Students

When inter-rater agreement was tested for 144 observations on Omnicam^®^-acquired 3D virtual models, the Fleiss Kappa results were statistically significant in all teeth (Table 5) for two different raters (numbers 7 and 9) with low experience levels (third-year dental students), and the results were statistically significant for Fleiss Kappa for all teeth (Table 5). The results were also statistically significant for lateral teeth alone.

#### 4.1.3. Inter-Rater Agreement Tests for Examiners with Different Experience Levels

When inter-rater agreement tests for examiners with different experience levels were performed for senior dentists scoring versus dental students, the values were very low, ranging between 0 and 0.184. However, there was a statistical significance (*p* < 0.05) for only lateral teeth, with an ICC equal to 0.184 (95% CI −0.022–0.375). Inter-rater agreement was tested for Omnicam^®^ for two different raters (numbers 3 and 7) with different experience levels (senior dentist and third-year dental student, respectively) for 144 observations, obtaining very low Cohen’s weighted Kappa and ICC values (<0.2) for lateral teeth and pits-and-fissures areas. The agreement tests were not in statistically significant agreement for all assessments except for lateral teeth (Table 6).

All intra- and inter-rater agreement tests performed for senior dentists yielded statistically significant results (*p* < 0.05).

## 5. Discussion

The present study has a similar methodology regarding ICDAS coding to that used in recent articles, particularly “Caries prevalence and caries index were established using ICDAS II 2–6/C-G and ICDAS II 4–6/E-G criteria for comparison with WHO indicators”, especially for white spot lesion assessments and monitoring [30].

When analyzing the limitations of the present study, a possible bias could be generated by the fact that the volunteers might have been students and interns with better theoretical and/or practical knowledge than their peers.

In a metanalysis published in 2021 on 51 articles, the authors calculated the following detection bias for DIAGNOdent^®^: “For permanent teeth, when histologic examination was considered as the reference for occlusal surfaces, the sensitivity (Se) range appeared high for the DIAGNOdent Pen (DD Pen) at 0.81–0.89, followed by ICDAS-II at 0.62–1, DIAGNOdent (DD) at 0.48–1, and bitewing radiography (BW) at 0–0.29” [2].

Besides dental impression acquisition, 3D virtual models are starting to be used for caries detection. For example, in a study published in 2022, the research team used intra- and inter-rater agreements, focusing on in vitro caries diagnosis on the 3D virtual models of extracted teeth. They aimed to compare three intraoral scanner-based caries diagnostic tools (Trios 4, iTero Element 5D, and Planmeca Emerald S) with the established methods (visual examination, bitewing radiography, and Diagnocam). Their null hypothesis was that there would be no difference between the caries diagnostic methods and the reference method, µ-CT, in terms of reliability (I), sensitivity and specificity (II), and logistic regression (III). The methodology of the above-mentioned study was extremely complex, relying on a gold standard, but only 64 teeth, primary molars, permanent premolars, and molars were included in that study, while incisors, canines, and crowned or filled teeth were excluded. They concluded that for proximal caries diagnoses of permanent teeth, Trios 4 and iTero Element 5D showed the same sensitivity, whereby the specificity of iTero Element 5D was higher. The highest specificity values were found for bitewing radiography, whereas the lowest values for sensitivity were observed in visual examination. Diagnocam demonstrated the highest sensitivity values [3].

For the presentation and comparison of our study’s results, we decided to use both Cohen and Fleiss kappa coefficients as well as inter-class correlations (ICCs) because repeated observations were recorded on the same subjects by different raters. To increase the statistical power, comparisons were made with a focus on a reduced degree of freedom: same examination method + same rater experience level + different patients; same patient + same rater + different examination methods. Hence, the number of observations for the comparison tests was slightly different (287 and 288, respectively) because there were two premolars extracted for orthodontic purposes, but the upper third molars were present. Therefore, we evaluated to what extent the examiner’s experience level influences the virtual model examination versus the clinical examination.

We chose the third-year dental students as part of the examiner team because they are just starting clinical examinations in their curricula. This choice was made to see to what extent examining color 3D virtual models is a reliable technique for accurate caries diagnosis.

Our findings are consistent with the easier diagnosis expected on lateral areas in previous studies [3]: for example, when we compared two different raters (numbers 3 and 7) with different experience levels (senior dentist and third-year dental student, respectively), only the ICC for lateral teeth and pits-and-fissures areas showed statistically significant agreement (*p* < 0.05), even though it was only <0.30, meaning a negligible correlation. For low-experience-level examiners, the best inter-rater agreements were obtained on lateral teeth and all teeth for Fleiss kappa coefficients, even though it only presented a negligible correlation (kappa < 0.30).

This is the first study comparing IOS 3D virtual models’ accuracy with ICDAS clinical examination for caries detection, to our knowledge. An increased number of clinical cases would help obtain a stronger statistical power of the study and higher correlation coefficients. In our study, the highest correlation coefficient obtained was an ICC of 0.969 (95% CI 0.949–0.981) for pits and fissures when senior dentists used the IOS Omnicam^®^. The examiners performed a careful professional cleaning before the scanning procedure to obtain well-cleaned, well-dried surfaces, or else the most versions of IOS software allow automatic filling in of the gaps in the 3D model, so information about caries might be difficult to retrieve when observing the 3D virtual models. Such a larger study would show if more intensive training of dental students and early-career dentists using color 3D virtual models could increase their inter-rater agreement, so a diagnosis on such virtual models can be recommended as part of their professional formation based on the fact that our study showed to what extent color virtual model examinations are sensitive to the examiner’s clinical experience, identifying which areas of the oral cavity raised more difficulties to less experienced raters. The less experienced examiners obtained lower inter-rater agreements, especially for smooth surfaces. Using any of the intraoral scanners tested in this study, the agreement was high for the most experienced raters, regardless of the examined area.

The observed discrepancies in the results, particularly in inter-rater agreement across different experience levels, highlight the impact of expertise on the utilization of advanced dental examination technologies. The excellent reliability noted among senior dentists when using Omnicam, as opposed to the moderate reliability observed with the Medit i500, can be explained because some devices, possibly Medit i500, may be more sensitive to the surrounding light, and the mirror system might be damaged sooner during sterilization. Thus, this may underscore the possible influence of user familiarity and proficiency with specific technological tools. Notably, the performance of Omnicam in capturing details of frontal and lateral teeth, as well as pits and fissures, was consistent among experienced practitioners, indicating a level of precision as a result of advanced ICDAS training and longer clinical experience. However, the stark contrast in poor agreement between students and between senior dentists and students using the same technology suggests a steep learning curve for caries diagnosis not related to the device itself but more likely related to their clinical knowledge. This emphasizes the need for comprehensive training and adaptation periods for less experienced practitioners. These findings align with our study’s objective to understand the reliability of modern dental examination technologies and their dependency on the operator’s expertise. They also point to a broader implication for the dental community: while advancing technologies promise enhanced diagnostic capabilities, their efficacy is closely tied to the user’s experience, necessitating focused educational strategies to bridge the gap between technology and examiner’s skills.

Our study shows the need to continuously adapt and update the university curriculum to keep up with new technologies. This intervention on the curriculum was also suggested by Elnawawy et al. who analyzed the ability of fifth- and sixth-year dental medicine students to correctly diagnose enamel caries based on the analysis of bitewing radiographs [31].

The study by Abreu-Placeres et al. had an interesting approach with data from students, university educators, and dentists from outside the academic environment regarding the management of carious risk and carious lesions. Since students and educators reported “performing ‘Caries detection and assessment’ 2D behaviors” more frequently than other dentists, the idea that there is a delay outside universities in the adoption of evidence-based dental medicine but also of differences in the training of doctors emerges [32]. Thus, there is a need to implement national, large-scale dental public health programs that include prevention programs and have, as a starting point, the training of all professionals in standardized systems for detecting and managing early carious lesions. Such programs would have an important impact in the long run, leading to an increase in the treatment of less extended caries, thus lowering the costs necessary for dental treatments (complex dental treatments lead to higher costs) over time and reducing the suffering of patients from dental causes, contributing to the increase in the dental health of the population and general well-being. Al Dhubayb’s cross-sectional study investigated the ability of 393 students and 100 dentists in private practice in Saudi Arabia to correctly detect carious lesions using the ICDAS system on color photographs of eight teeth. The participants had the greatest difficulties in recognizing the right code for enamel lesions with ICDAS codes 1 and 2 [33]. In the above-mentioned study, the mean percentage ability score to detect caries using ICDAS for third-year students and general practitioners was 38.6% and 38.7%, respectively. The values obtained in our study are in line with the previously cited study for the inter-rater agreement of third year dental students, which we found to have a maximum of 0.275 (Fleiss’ kappa for pits and fissures). In our study, the majority of the carious lesions had an ICDAS score of 1 or 2, so the color 3D virtual model method can be used as a complementary procedure in these clinical situations. Moreover, the intra- and inter-rater agreements for dental students were slight in most cases (values < 0.20) and fair (values between 0.21 and 0.40) for pits-and-fissures Fleiss kappa values. On the other hand, inter-rater agreement for senior dentists was substantial (values between 0.61 and 0.80) for frontal teeth, where Fleiss kappa was 0.659, and for smooth surfaces, where Cohen’s weighted Kappa was 0.663. Senior dentists’ agreement was almost perfect for all the other agreement tests performed on scores assigned by examining Omnicam-recorded 3D virtual models. This very good agreement of senior dentists may be explained by their more than 5 years of clinical experience combined with recent hands-on training using ICDAS. This method can be useful for students or early career dentists who, in certain situations, would like another opinion on the diagnosis; thus, they can send the color 3D scan in the .ply format to a mentor or a practitioner with more experience [34].

Color virtual 3D models are easier and faster to examine compared to traditional clinical examination. They could be used for teledentistry consultations (e.g., dental hygienists record the IOS 3D models and send them to the dentists for remineralization therapy success analysis). In the near future one could even expect some AI-based software employing fog computing to be developed for dental caries diagnosis and management. This would allow a real-time feedback of the remineralization therapy efficiency, because treatment needs continue to increase, and it is highly important to have an accurate overview of dental status [35].

“An AI-based analytics solution leverages clustering and correlation algorithms to provide a root-cause analysis so that any issues can be remediated as soon as possible” [36]. Such an approach would allow real-time correlations and corrections, if necessary, of intraoral scanning data, clinical examinations (ideally video-recorded while the dentist is wearing magnification and camera), and DIAGNOdent recordings. Hence, one can obtain an accurate diagnosis from three different assessment methods for early-stage caries. Using any of the three types of intraoral scanners tested in this study, the good correlation with clinical examination may allow warning messages to be generated in order to re-evaluate the dental areas for which significant discrepancies appear between different diagnostic methods and in the future with the help of AI. All of this will contribute to increasing the accuracy of early-stage caries diagnosis. Our study also brings important data from the perspective of minimally invasive dentistry since obtaining IOS 3D models is non-radiative compared to dental radiographs, a particularly important element when considering children or pregnant women. Also, sometimes, little patients have a lower compliance: they move during the exposure and the exposure must be repeated, thus increasing the dose of radiation captured. With digital models, this problem no longer occurs, and our study demonstrates that the technique of diagnosing early caries using IOS 3D models is a reliable one, provided that the examiner has acquired the experience and skills to use each type of oral scanner.

## 6. Conclusions

Intra-rater and inter-rater agreement tests and interclass correlations showed statistically significant results (*p* < 0.001) for senior dentists: intra-rater agreement between Omnicam and clinical examination by senior dentists had excellent reliability, while Medit i500 had moderate reliability. For senior dentists, the inter-rater agreement for Omnicam was excellent for frontal and lateral teeth, while it was moderate for pits and fissures. Using Omnicam, the inter-rater agreement between students and between senior dentists and students was poor, while the inter-rater agreement between senior dentists was excellent.

We conclude that Omnicam may become a reliable method for second-opinion diagnosis and overview of dental treatment success. Our study opened a new perspective in the use of color 3D virtual dental models (.ply format), but other intraoral scanning systems must also be investigated.

## Figures and Tables

**Table 1:** Synopsis of examinations analyzed.

Rater’s Experience Level	Number of Surfaces Rated by Clinical Examinations	Number of Surfaces Rated by Examining with IOS Medit i500^®^	Number of Surfaces Rated by Examining with IOS Omnicam^®^
third-year dental students	144	287	288
senior dentists (more than 5 years of clinical experience)	575	287	576

**Table 2:** Intra-rater agreement for Medit i500^®^ with clinical examination.

Observations	Number Observations	Cohen’s Weighted Kappa	*p*-Value	Fleiss Kappa	*p*-Value	ICC (95% CI)	*p*-Value
All teeth	287	0.608	<0.001	0.58	<0.001	0.614 (95% CI 0.536–0.681)	<0.001
Frontal teeth	104	0.493	<0.001	0.593	<0.001	ICC = 0.368 (95% CI 0.191–0.522	<0.001
Lateral teeth	183	0.634	<0.001	0.575	<0.001	ICC = 0.663 (95% CI 0.573–0.737)	<0.001
Pits and fissures	63	0.586	<0.001	0.548	<0.001	ICC = 0.592 (95% CI 0.405–0.732)	<0.001
Smooth surface	224	0.568	<0.001	0.597	<0.001	ICC = 0.543 (95% CI 0.444–0.629)	<0.001

CI, confidence interval; ICC, interclass correlation coefficient.

**Table 3:** Intra-rater agreement values for rater no. 3 (senior dentist) between clinical and IOS.O.

Observations	Number Observations	Cohen’s Weighted Kappa	*p*-Value	Fleiss Kappa	*p*-Value	ICC (95% CI)	*p*-Value
All teeth	288	0.863	<0.001	0.771	<0.001	0.921 (95% CI 0.902–0.937)	<0.001
Frontal teeth	104	0.829	<0.001	0.659	<0.001	0.922 (95% CI 0.887–0.946)	<0.001
Lateral teeth	184	0.872	<0.001	0.802	<0.001	0.921 (95% CI 0.896–0.941)	<0.001
Pits and fissures	64	0.895	<0.001	0.795	<0.001	0.955 (95% CI 0.926–0.972)	<0.001
Smooth surface	224	0.663	<0.001	0.596	<0.001	0.704 (95% CI 0.631–0.764)	<0.001

CI, confidence interval; ICC, interclass correlation coefficient.

**Table 4:** Inter-rater agreement tests for 288 observations recorded by senior dentists using Omnicam^®^.

Observations	Number Observations	Cohen’s Weighted Kappa	*p*-Value	Fleiss Kappa	*p*-Value	ICC (95% CI)	*p*-Value
All teeth	288	0.902	<0.001	0.809	<0.001	0.959 (95% CI 0.948–0.967)	<0.001
Frontal teeth	104	0.829	<0.001	0.659	<0.001	0.922 (95% CI 0.887–0.946)	<0.001
Lateral teeth	184	0.924	<0.001	0.852	<0.001	0.968 (95% CI 0.958–0.976	<0.001
Pits and fissures	64	0.923	<0.001	0.846	<0.001	0.969 (95% CI 0.949–0.981)	<0.001
Smooth surface	224	0.748	<0.001	0.597	<0.001	0.856 (95% CI 0.817–0.888)	<0.001

CI, confidence interval; ICC, interclass correlation coefficient.

**Table 5:** Inter-rater agreement IOS.O.7.cu.9.

Observations	Number Observations	Cohen’s Weighted Kappa	*p*-Value	Fleiss Kappa	*p*-Value	ICC (95% CI)	*p*-Value
All teeth	144	0.106	0.084	0.179	0.004	0.04 (95% CI −0.124–0.201)	0.317
Frontal teeth	52	0	1	−0.02	0.888	0 (95% CI −0.264–0.267)	0.5
Lateral teeth	92	0.171	0.051	0.223	0.011	0.054 (95% CI −0.154–0.256)	0.306
Pits and fissures	32	0.223	0.135	0.275	0.078	0.084 (95% CI −0.276–0.419)	0.325
Smooth surface	112	0	1	−0.012	0.856	0 (95% CI −0.18–0.181)	0.5

CI, confidence interval; ICC, interclass correlation coefficient.

**Table 6:** Omnicam^®^ inter-rater agreement between 2 different raters of different experience levels (senior dentist versus a third-year dental student: observers 3 and 7) for 144 observations.

Observations	Number Observations	Cohen’s Weighted Kappa	*p*-Value	Fleiss Kappa	*p*-Value	ICC (95% CI)	*p*-Value
All teeth	144	0.114	0.114	0.077	0.23	0.126 (95% CI −0.037–0.284)	0.065
Frontal teeth	52	0	1	−0.02	0.888	0 (95% CI −0.264–0.267)	0.5
Lateral teeth	92	0.155	0.051	0.094	0.279	0.184 (95% CI −0.022–0.375)	0.04
Pits and fissures	32	0.1	0.449	0.028	0.845	0.128 (95% CI −0.238–0.456)	0.245
Smooth surface	112	0	1	−0.009	0.924	0 (95% CI −0.183–0.183)	0.5

CI, confidence interval; ICC, interclass correlation coefficient.

## Data Availability

Data will be made available upon request due to privacy and ethical restrictions.

## Supplementary Materials

The following supporting information can be downloaded at https://www.mdpi.com/article/10.3390/medicina59122157/s1, Figure S1. Virtual 3D model opened in Exocad Viewer.

## References

[1] World Health Organization Oral Health. https://www.who.int/news-room/fact-sheets/detail/oral-health/.

[2] Foros P., Oikonomou E., Koletsi D., Rahiotis C. (2021). Detection Methods for Early Caries Diagnosis: A Systematic Review and Meta-Analysis. Caries Res..

[3] New Caries Diagnostic Tools in Intraoral Scanners: A Comparative In Vitro Study to Established Methods in Permanent and Primary Teeth—PMC. https://www.ncbi.nlm.nih.gov/pmc/articles/PMC8950989/.

[4] Srilatha A., Doshi D., Kulkarni S., Reddy M.P., Bharathi V. (2020). Advanced Diagnostic Aids in Dental Caries—A Review. JGOH.

[5] Sandhu M., Gupta N., Jhingan P. (2019). Comparison of Visual Examination and Magnification with DIAGNOdent for Detection of Smooth Surface Initial Carious Lesion–Dry and Wet Conditions. Int. J. Clin. Pediatr. Dent..

[6] Braga M.M., Mendes F.M., Ekstrand K.R. (2010). Detection Activity Assessment and Diagnosis of Dental Caries Lesions. Dent. Clin. N. Am..

[7] Kühnisch J., Dietz W., Stösser L., Hickel R., Heinrich-Weltzien R. (2007). Effects of Dental Probing on Occlusal Surfaces—A Scanning Electron Microscopy Evaluation. Caries Res..

[8] Baltacioglu I.H., Orhan K. (2017). Comparison of Diagnostic Methods for Early Interproximal Caries Detection with Near-Infrared Light Transillumination: An in Vivo Study. BMC Oral Health.

[9] Muñoz-Sandoval C., Gambetta-Tessini K., Botelho J.N., Giacaman R.A. (2022). Detection of Cavitated Proximal Carious Lesions in Permanent Teeth: A Visual and Radiographic Assessment. Caries Res..

[10] Govind S., Jena A., Kamilla S.K., Mohanty N., Mallikarjuna R.M., Nalawade T., Saraf S., Khaldi N.A., Jahdhami S.A., Shivagange V. (2023). Diagnosis and Assessment of Dental Caries Using Novel Bioactive Caries Detecting Dye Solution. Biomedicines.

[11] Shi X.-Q., Welander U., Angmar-Månsson B. (2000). Occlusal Caries Detection with KaVo DIAGNOdent and Radiography: An in Vitro Comparison. Caries Res..

[12] Tranaeus S., Lindgren L.-E., Karlsson L., Angmar-Månsson B. (2004). In Vivo Validity and Reliability of IR Fluorescence Measurements for Caries Detection and Quantification. Swed. Dent. J..

[13] Hung H.V., Ngoc V.T.N., Vu Thi H., Chu D.-T. (2021). Early Childhood Caries in Obese Children: The Status and Associated Factors in the Suburban Areas in Hanoi, Vietnam. Int. J. Environ. Res. Public Health.

[14] Serban C., Lungeanu D., Bota S.D., Cotca C.C., Negrutiu M.L., Duma V.F., Sinescu C., Craciunescu E.L. (2022). Emerging Technologies for Dentin Caries Detection-A Systematic Review and Meta-Analysis. J. Clin. Med..

[15] Luczaj-Cepowicz E., Marczuk-Kolada G., Obidzinska M., Sidun J. (2019). Diagnostic Validity of the Use of ICDAS II and DIAGNOdent Pen Verified by Micro-Computed Tomography for the Detection of Occlusal Caries Lesions—An in Vitro Evaluation. Lasers Med. Sci..

[16] Kockanat A., Unal M. (2017). In Vivo and in Vitro Comparison of ICDAS II, DIAGNOdent Pen, CarieScan PRO and SoproLife Camera for Occlusal Caries Detection in Primary Molar Teeth. Eur. J. Paediatr. Dent..

[17] Melo M., Sanz J.L., Forner L., Rodríguez-Lozano F.J., Guerrero-Gironés J. (2022). Current Status and Trends in Research on Caries Diagnosis: A Bibliometric Analysis. Int. J. Environ. Res. Public Health.

[18] Chan E.K., Wah Y.Y., Lam W.Y.-H., Chu C.-H., Yu O.Y. (2023). Use of Digital Diagnostic Aids for Initial Caries Detection: A Review. Dent. J..

[19] Witecy C., Ganss C., Wöstmann B., Schlenz M.B., Schlenz M.A. (2021). Monitoring of Erosive Tooth Wear with Intraoral Scanners In Vitro. Caries Res..

[20] Steinmeier S., Wiedemeier D., Hämmerle C.H.F., Mühlemann S. (2020). Accuracy of Remote Diagnoses Using Intraoral Scans Captured in Approximate True Color: A Pilot and Validation Study in Teledentistry. BMC Oral Health.

[21] Mangano F., Gandolfi A., Luongo G., Logozzo S. (2017). Intraoral Scanners in Dentistry: A Review of the Current Literature. BMC Oral Health.

[22] Michou S., Lambach M.S., Ntovas P., Benetti A.R., Bakhshandeh A., Rahiotis C., Ekstrand K.R., Vannahme C. (2021). Automated Caries Detection in Vivo Using a 3D Intraoral Scanner. Sci. Rep..

[23] Duong D.L., Kabir M.H., Kuo R.F. (2021). Automated Caries Detection with Smartphone Color Photography Using Machine Learning. Health Inform. J..

[24] Ntovas P., Michou S., Benetti A., Bakhshandeh A., Ekstrand K., Rahiotis C., Kakaboura A. (2023). Occlusal Caries Detection on 3D Models Obtained with an Intraoral Scanner. A Validation Study. J. Dent..

[25] Dikmen B. (2015). Icdas II Criteria (International Caries Detection and Assessment System). J. Istanbul Univ. Fac. Dent..

[26] International Caries Detection and Assessment System (ICDAS) Coordinating Committee Rationale and Evidence for the International Caries Detection and Assessment System (ICDAS II). Proceedings of the Workshop.

[27] International Caries Detection and Assessment System (ICDAS) Coordinating Committee Criteria Manual-International Caries Detection and Assessment System (ICDAS II). Proceedings of the Workshop.

[28] Transparent Reporting of a Multivariable Prediction Model for Individual Prognosis or Diagnosis (TRIPOD): The TRIPOD Statement|EQUATOR Network. https://www.equator-network.org/reporting-guidelines/tripod-statement/.

[29] R Core Team (2023). R: A Language and Environment for Statistical Computing.

[30] Ondine Lucaciu P., Mester A., Constantin I., Orban N., Cosma L., Candrea S., Sava-Rosianu R., Mesaros A.S. (2020). A WHO Pathfinder Survey of Dental Caries in 6 and 12-Year Old Transylvanian Children and the Possible Correlation with Their Family Background, Oral-Health Behavior, and the Intake of Sweets. Int. J. Environ. Res. Public Health.

[31] Elnawawy M.S.A., Gharote H. (2022). Dental Students’ Ability to Detect Only-Enamel Proximal Caries on Bitewing Radiographs. Cureus.

[32] Abreu-Placeres N., Newton J.T., Avila V., Garrido L.E., Jácome-Liévano S., Pitts N.B., Ekstrand K.R., Ochoa E.M., Martignon S. (2023). How Do Dental Practitioners, Educators and Students Diagnose and Manage Caries Risk and Caries Lesions? A COM-B Analysis. Community Dent. Oral Epidemiol..

[33] Al Dhubayb S., Al Sultan M., Al Sudairi S., Hakami F., Al Sweleh F.S. (2021). Ability of Dentists and Students to Detect Caries by Using the International Caries Detection and Assessment System. CCIDE.

[34] Algarni A.A., Alwusaydi R.M., Alenezi R.S., Alharbi N.A., Alqadi S.F. (2023). Knowledge and attitude of dentists toward minimally invasive caries management in Almadinah Almunawwarah province, KSA. J. Taibah Univ. Med. Sci..

[35] Ahmed E.E.A., Nesser S.A., Schmoeckel J. (2023). Introducing an Innovative Approach for Managing Proximal Non-Cavitated Carious Lesions in Juvenile Permanent Dentition: Combining Orthodontic Separators and Silver Fluoride Application. Medicina.

[36] What Is AI Analytics?. https://www.anodot.com/learning-center/ai-analytics/.

