# Supplementary figures and images for: Caries Lesion Assessment Using 3D Virtual Models by Examiners with Different Degrees of Clinical Experience
Source: Medicina (Kaunas). 2023 Dec 13;59(12):2157. doi: 10.3390/medicina59122157 (PMC10744345; doi:10.3390/medicina59122157)

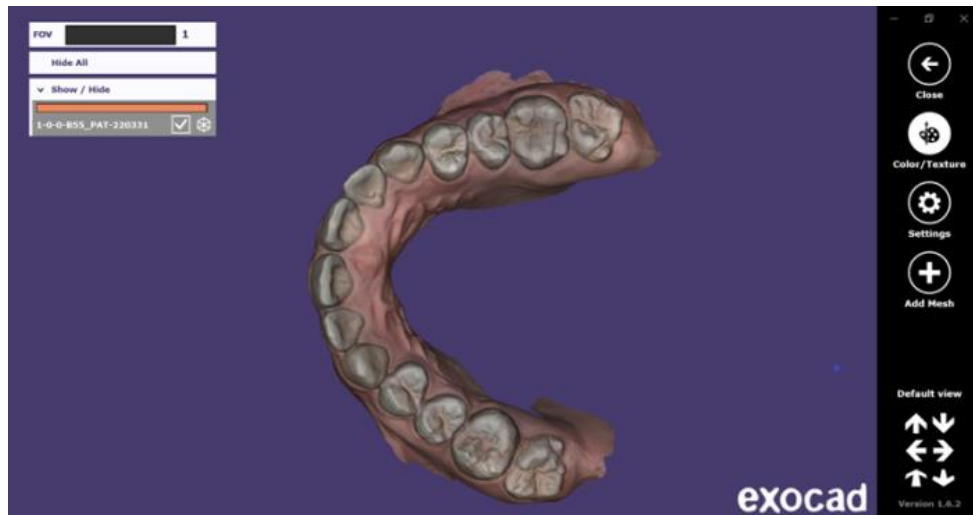

**Figure S1.** Virtual 3D model opened in Exocad Viewer.

Supplement: Supplementary file 1 [file medicina-59-02157-s001.zip › medicina-2737316-supplementary.pdf]
